# Transaminases for industrial biocatalysis: novel enzyme discovery

**DOI:** 10.1007/s00253-020-10585-0

**Published:** 2020-04-16

**Authors:** Stephen A. Kelly, Stefan Mix, Thomas S. Moody, Brendan F. Gilmore

**Affiliations:** 1 School of Pharmacy, Queen’s University Belfast, Belfast, BT9 7BL Northern Ireland; 2 Department of Biocatalysis & Isotope Chemistry, Almac, 20 Seagoe Industrial Estate, Craigavon, UK; 3 Arran Chemical Company Limited, Unit 1 Monksland Industrial Estate, Athlone, Co. Roscommon Ireland

**Keywords:** Biocatalysis, Chiral amine, Enzyme discovery, Metagenomics, Transaminase

## Abstract

**Abstract:**

Transaminases (TAms) are important enzymes for the production of chiral amines for the pharmaceutical and fine chemical industries. Novel TAms for use in these industries have been discovered using a range of approaches, including activity-guided methods and homologous sequence searches from cultured microorganisms to searches using key motifs and metagenomic mining of environmental DNA libraries. This mini-review focuses on the methods used for TAm discovery over the past two decades, analyzing the changing trends in the field and highlighting the advantages and drawbacks of the respective approaches used. This review will also discuss the role of protein engineering in the development of novel TAms and explore possible directions for future TAm discovery for application in industrial biocatalysis.

**Key Points:**

*• The past two decades of TAm enzyme discovery approaches are explored.*

*• TAm sequences are phylogenetically analyzed and compared to other discovery methods.*

*• Benefits and drawbacks of discovery approaches for novel biocatalysts are discussed.*

*• The role of protein engineering and future discovery directions is highlighted.*

## Introduction

Transaminases (TAms) are increasingly important enzymes for the pharmaceutical and fine chemical industries, capable of producing valuable chiral amine drug intermediates from a prochiral precursor in a single step (Ghislieri and Turner 2013; Kelly et al. 2018a).

TAms have a number of advantages over conventional chemical synthesis of chiral amines, including excellent stereoselectivity, as well as an ability to perform under mild conditions, replacement of often toxic transition metal catalysts as alternative technology, and reduction of the use of volatile organic solvents (VOCs) in chemical manufacturing. The best known example of TAm application in the pharmaceutical industry is in the production of the antidiabetic drug sitagliptin (Savile et al. 2010). In this case, a combination of in silico design and directed evolution were used to engineer the enzyme’s large binding pocket, improving activity towards its ketone substrate, prositagliptin, and improving stability to the conditions required for substrate solubilization. The final mutant resulted in an overall yield increase of 10–13%, 53% increase in productivity and 19% reduction in total waste, compared to the use of chemical methods. The TAm-catalyzed reaction negated the need for a toxic transition metal catalyst and expensive high-pressure hydrogenation equipment, conferring further advantages over the conventional approach.

Biocatalysis has been increasingly employed to improve and streamline chemical syntheses, increasing with it the demand for new and enhanced enzymes for application in these processes. As a result, novel TAms continue to be discovered, from a wide variety of sources using different discovery methods.

There have been a number of excellent TAm reviews written in recent years, focusing on a range of topics including classification, application, engineering, and optimization of TAms and their reactions (Guo and Berglund 2017; Ferrandi and Monti 2018; Gomm and O’Reilly 2018; Kelly et al. 2018a; Patil et al. 2018). This mini-review will focus on the approaches used in novel wild-type TAm discovery over the past two decades, highlighting the changing trends in the field during this time. It will also scrutinize the benefits and drawbacks of the various discovery approaches and provide comment on possible directions for future TAm discovery for application in industrial biocatalysis.

## Culture-based enzyme discovery

### Activity-guided enzyme discovery

The first examples of TAm discovery and exploitation for biocatalysis were from cultured soil bacteria. This work was largely pioneered by Shin and Kim in the late 1990s and early 2000s, with early examples including TAms from *Klebsiella pneumoniae* JS2F and *Bacillus thuringiensis* JS64 (Shin and Kim 1997). Enrichment cultures using 1-phenylethylamine (1-PEA) as sole nitrogen source were used to identify organisms harboring a TAm capable of metabolizing this amine. This culture-based approach yielded one of the most well-characterized TAms, an (*S*)-selective enzyme from *Vibrio fluvialis* (*Vf*-TAm) (Shin and Kim 1999). Much of what is understood about TAms today comes from investigations involving *Vf-*TAm, including the two-site binding pocket architecture of TAms, which was proposed based on observations of this enzyme (Shin and Kim 2002).

Shifting the equilibrium of the TAm-catalyzed reaction to enhance product formation was also investigated using *Vf*-TAm, through the use of L-alanine as an amino donor and removal of the pyruvate co-product using lactate dehydrogenase (LDH) (Shin and Kim 1999). Alleviating product inhibition with alternative amino donors was investigated using *Vf-*TAm, by employing a biphasic system to remove the benzaldehyde co-product in a reaction which produced an intermediate for the anti-epilepsy drug levetiracetam (Shin and Kim 2009). *Vf*-TAm has been used in the synthesis of a number of other pharmaceutical intermediates, including (*S*)-rivastigmine, a drug used in Alzheimer’s disease and dementia associated with Parkinson’s disease (Fuchs et al. 2010). In this example, pyruvate removal was facilitated through the use of an LDH-based system outlined above, further increasing the efficiency of the process. The same research group combined *Vf*-TAm in a cascade reaction with a galactose oxidase enzyme, affording an amine intermediate for the potent antifungal drug naftifine, using an alcohol starting material (Fuchs et al. 2012)

Following the initial success of work by Shin and Kim, a number of other *S*-selective TAms were discovered using enrichment media in a culture-dependent approach, including those from *Alcaligenes denitrificans* Y2k-2 (*Ad*-TAm) (Yun et al. 2004), *Mezorhizobium* sp. LUK (*Mz*-TAm) (Kim et al. 2007), and *Bacillus megaterium* SC6394 (*Bm*-TAm) (Hanson et al. 2008). *Bm*-TAm has been used in the synthesis of (*R*)-*sec*-butylamine and (*R*)-1-cyclopropylethylamine, important intermediates for corticotropin releasing factor (CRF-1) antagonists (Hanson et al. 2008), which have been proposed for the treatment of depression and anxiety.

Other variations of culture-dependent TAm mining have been applied successfully for the discovery of *S*-selective TAms, including gas chromatography-based screening of whole-cell preparations for the ability to convert 1-PEA (Clay et al. 2010) and metabolic function analysis (Lang et al. 2009; Weber et al. 2014)

The first *R*-selective TAm was also discovered using an activity-guided approach. An ω-TAm from *Arthrobacter* sp. KNK168 (*Arth*-TAm) was isolated from soil bacteria using enrichment media containing 3,4-dimethoxyamphetamine as the sole nitrogen source (Iwasaki et al. 2003). This enzyme, and subsequent mutated variants, has had a profound effect on the application of TAms in pharmaceutical production. Protein engineered mutants of *Arth*-TAm have been applied in the asymmetric synthesis of the dual orexin receptor antagonist, suvorexant (Mangion et al. 2012), of silodosin, a pro-drug for the treatment of benign prostatic hypertrophy (Simon et al. 2014), and the anti-arrhythmic, mexiletine (Koszelewski et al. 2009), among others. Indeed, the most highly publicized and successful application of TAm biocatalysis was using an engineered variant of *Arth*-TAm, in the synthesis of the antidiabetic drug sitagliptin, an example highlighted previously (Savile et al. 2010).

Discovery of *R*-selective wild-type TAms using activity-guided approaches has resulted in limited success, with only a handful of such enzymes uncovered in this way. Using *R*-amines as the sole nitrogen source in enrichment media, Pavkov-Keller and co-workers discovered and characterized *R*-selective TAms from *Curtobacterium pusillum* and *Microbacterium ginsengisoli* (Pavkov-Keller et al. 2016). These enzymes were not easily classified along with previously known TAms, given their structural similarity to D-amino acid TAms and branched-chain amino acid TAms, combined with their ability to accept amines as substrates. As such, this study forced a rethink about PLP fold-type IV TAms, and to reassess what is known about structure-function relationships in this group. This would not have been possible without the biochemical characterization following activity-guided discovery, once again highlighting the benefits of this approach.

**Fig. 1:**
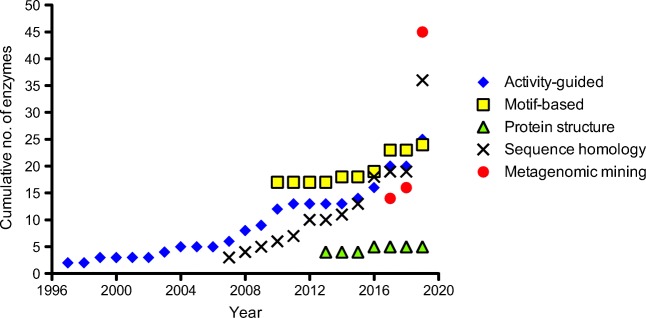
Cumulative number of reported TAms, grouped by discovery approach from 1997 to 2019

Activity-guided enzyme discovery from cultured microorganisms remained the gold standard for uncovering novel enzymes for several years following its initial successes. While a number of other discovery approaches have emerged in recent years, such as culture-independent metagenome screening, novel enzymes continue to be discovered in this way (Fig. 1) (Wilding et al. 2015, 2016; Wu et al. 2017; Noshahri et al. 2019). In the latter example by Noshahri et al., the decades-old approach of using enrichment media with 1-PEA as the sole nitrogen source was given a new lease of life by applying this method to microorganisms from challenging environments. TAms from *Bacillus halotolerans* and *Bacillus subtilis* subsp. *stercoris* were isolated from a petroleum refinery and an oil field respectively. The resulting enzymes showed tolerance to organic solvents, performing best in 30% DMSO, as well as an acidophilic profile (optimum activity at pH 5, with 70% activity remaining at pH 3) (Noshahri et al. 2019). Recent examples like this serve to reinforce the benefits of continuing to use activity-guided methods for enzyme discovery, in particular when the advantages of this approach can be extended to previously untapped and extreme environments. TAms discovered using activity-guided approaches, as well as those discovered from other culture-dependent methods, are summarized in Table 1.

**Table 1:** Summary of TAms discovered from cultured microorganisms

Source organism	(*S*)/(*R*)	Comments	Reference
Activity-guided approach			
*Klebsiella pneumoniae* JS2F	(*S*)	Enrichment media used with 1-PEA as SNS	Shin and Kim (1997)
*Bacillus thuringiensis* JS64	(*S*)		
*V. fluvialis* JS17	(*S*)	Enrichment media used with 1-PEA as SNS	Shin and Kim (1999) Shin et al. (2001)
*Arthrobacter* sp. KNK168	(*R*)	Enrichment media with 3,4-dimethoxyamphetamine as SNS	Iwasaki et al. (2003) Iwasaki et al. (2012)
*Pseudomonas* sp. KNK425	(*S*)		
*Alcaligenes denitrificans* Y2k-2	(*S*)	Enrichment media with β-amino-*n*-butyric acid as SNS	Yun et al. (2004)
*Mesorhizobium* sp. LUK	(*S*)	Enrichment media with a substituted β-amino-3-phenylpropionic acid	Kim et al. (2007)
*Bacillus megaterium* SC6394	(*S*)	Enrichment media used with 1-cyclopropylethylamine as SNS	Hanson et al. (2008)
*Moraxella lacunata* WZ34	(*S*)	Enrichment media used with alaninol as SNS	Chen et al. (2008)
*Capsicum*	(*S*)	Discovered through metabolic function analysis	Lang et al. (2009); Weber et al. (2014)
*Janibacter terrae* DSM13953	(*S*)	GC-based assay screening whole cell preparations against 1-PEA	Clay et al. (2010)
*Pseudomonas cichorii* DSM 50259	(*S*)		
*P seudomonas fluorescens* ATCC49838	(*S*)		
*Pseudomonas fluorescens* KNK08–18	(*S*)	Enrichment media with 7-methoxy-2-aminotetraline as SNS	Ito et al. (2011)
*Pseudomonas* sp. ACC	(*S*)	Enrichment media used with 12-aminododecanoic acid as SNS	Wilding et al. (2015); Wilding et al. (2016)
*Curtobacterium pusillum*	(*R*)		Pavkov-Keller et al. (2016)
*Microbacterium ginsengisoli*	(*R*)		
*Pseudomonas putida* NBRC14164	(*S*)	Enrichment media with 1-PEA as SNS	Wu et al. (2017)
*Bacillus halotolerans*	(*S*)	Enrichment media with 1-PEA as SNS Strain isolated from a petroleum refinery	Noshahri et al. (2019)
*Bacillus subtilis* subsp. *stercoris*	(*S*)	Enrichment media used with 1-PEA as SNS Strain isolated from an oil field	
*Bacillus subtilis* subsp. *inaquosorum*	(*S*)		
*Bacillus endophyticus*	(*S*)		
*Rhizobium radiobacter*	(*S*)		
Sequence homology			
*Chromobacterium violaceum* DSM30191	(*S*)	Homologous sequence searching with *Vf*-TAm	Kaulmann et al. (2007)
*Pseudomonas aeruginosa*	(*S*)		Ingram et al. (2007)
*Arthrobacter citreus*	(*S*)		Martin et al. (2007)
*Caulobacter crescentus*	(*S*)	Homologous sequence searching with *A. denitrificans* TAm	Hwang et al. (2008)
*Rhodobacter sphaeroides*	(*S*)		Schätzle et al. (2009)
*Paracoccus denitrificans*	(*S*)	Homologous sequence searching with *Vf-*TAm	Park et al. (2010)
*Polaromonas* sp. JS666	(*S*)	Homologous sequence searching with *Mesorhizobium* TAm	Bea et al. (2011)
*Ochrobactrum anthropi*	(*S*)	Homologous sequence searching with *Pd-*TAm	Park et al. (2012)
*Acinetobacter baumannii*	(*S*)		
*Acetobacter pasteurianus*	(*S*)		
*Burkholderia vietnamensis*	(*S*)	Homologous sequence searching with *Vf*-TAm	Jiang et al. (2014)
*Halomonas elongata*	(*S*)	Homologous sequence searching with *Vf-* TAm First TAm characterized from halophilic bacteria	Cerioli et al. (2015)
*Burkholderia graminis*	(*S*)		Mathew et al. (2015)
*Thermomicrobium roseum*	(*S*)	From thermophilic bacterium	Mathew et al. (2016a)
*Sphaerobacter thermophilus*	(*S*)	Homologous sequence searching with *Polaromonas-*TAm From thermophilic bacterium	Mathew et al. (2016b)
*Geobacillus thermodenitrificans*	(*S*)	Homologous sequence searching with *Vf-* TAm From thermophilic bacterium	Chen et al. (2016)
*Bacillus megaterium*	(*S*)	Homologous sequence searching with EcK12*-*TAm	Slabu et al. (2016)
*Bacillus mycoides*	(*S*)	Homologous sequence searching with EcK12*-*TAm	Slabu et al. (2016)
*Fusarium oxysporum*	(*R*)		Gao et al. (2017)
*Halomonas* sp. CSM-2	(*S*)		Kelly et al. (2017)
*Halorubrum* sp. CSM-61	(*R*)	First haloarchaeal TAm characterized for biocatalysis	Kelly et al. (2019a, b)
*Rhodospirillaceae bacterium*	(*S*)	Homologous sequence searching with *Polaromonas*-TAm	Kim et al. (2019a, b)
*Labrenzia* sp. LAB	(*S*)		
*Afipia* sp. P52–10	(*S*)		
*Oceanibaculum indicum*	(*S*)		
*Ilumatobacter coccineus*	(*S*)		
*Variovorax* sp. KK3	(*S*)		
*Paraburkholderia caribensis*	(*S*)		
*Hydrogenophaga palleronii*	(*S*)		
*Solirubrobacter soli*	(*S*)	Homologous sequence searching with *Ilumatobacter*-TAm	
*Kineosporia* sp. R_H_3	(*S*)		
*Roseomonas deserti*	(*S*)		
*Sinorhizobium meliloti*	(*S*)		
*Bosea lupine*	(*S*)		
*Bosea vaviloviae*	(*S*)		
*Pseudacidovorax intermedius*	(*S*)		
*Burkholderia* sp. UYPR1.413	(*S*)		
Key motif-based search			
*Aspergillus terreus*	(*R*)	Fungal source	Höhne et al. (2010)
*Penicillium chrysogenum*	(*R*)		
*Aspergillus oryzae*	(*R*)		
*Aspergillus fumigatus*	(*R*)		
*Neosartorya fischeri*	(*R*)		
*Gibberella zeae*	(*R*)		
*Hyphomonas neptunium*	(*R*)	Isolated from seawater	
*Mycobacterium vanbaalenii*	(*R*)	Actinobacteria First isolated from petroleum-contaminated estuarine sediments	
*Mesorhizobium loti* 1	(*R*)		
*Mesorhizobium loti* 2	(*R*)		
*Marinomonas* sp.	(*R*)		
*Rhizobium etli*	(*R*)		
*Rhodoferax ferrireducens*	(*R*)		
*Jannashcia* sp.	(*R*)		
*Labrenzia alexandrii*	(*R*)		
*Burkholderia* sp.	(*R*)		
Gamma proteobacterium	(*R*)		
*Nectria haematococca*	(*R*)	Fungal source	Sayer et al. (2014)
*Escherichia coli* K12	(*S*)	*ygjG* gene	Slabu et al. (2016)
*Capronia semiiersa*	(*R*)		Iglesias et al. (2017)
*Pseudomonas putida*	(*S*)		Galman et al. (2017)
*Pseudomonas fluorescens*	(*S*)		
*Pseudomonas chlororaphis*	(*S*)		
*Actinobacter* sp.	(*R*)		Tang et al. (2019)
Protein structure-based search			
*Silicibacter pomeroyi*	(*S*)		Steffen-Munsberg et al. (2013)
*Rhodobacter sphaeroides* KD131	(*S*)		
*Ruegeria* sp. TM1040	(*S*)		
*Mesorhizobium loti* MAFF30399	(*S*)		
*Bacillus anthracis*	(*S*)		Steffen-Munsberg et al. (2016)

*SNS* sole nitrogen source

### Homologous sequence searching

The advent of cost-effective genome sequencing, and the subsequent effect this has had in bolstering sequence databases, has transformed the way we search for new enzymes. Searching for TAm sequences homologous to those uncovered using activity-guided approaches has massively increased the speed at which new functional sequences can be found and allows us to benefit from the vast sequence data available in both private and public databases.

Unsurprisingly, given its early successes, homologs were sought for *Vf*-TAm. Using the archetypal *Vf*-TAm sequence as a template, ω-TAm from *Chromobacterium violaceum* (*Cv*-TAm) was discovered and characterized (Kaulmann et al. 2007). *Cv*-TAm remains one of the most characterized and utilized TAms and has been employed in the synthesis of a number of pharmaceutical intermediates, including the thromboxane receptor antagonist, ramatroban (Busto et al. 2014), and the decongestant, norephedrine (Sehl et al. 2013).

As outlined previously, *Vf*-TAm was employed by Fuchs et al. in the synthesis of the cholinesterase inhibitor, rivastigmine. The same group discovered a novel TAm from *Paracoccus denitrificans* (*Pd*-TAm) based on sequence homology to *Vf*-TAm and was able to use this new enzyme to improve the biocatalytic process used in rivastigmine synthesis (Fuchs et al. 2010).

Following successful homology searches using enzymes discovered with an activity-guided approach, a natural progression in TAm discovery was to use these newly discovered enzymes as templates in further homologous sequence searches. The use of *Pd*-TAm as a template sequence yielded a number of novel TAms (Park et al. 2012). One of these enzymes from *Ochrobacterium anthropi* (*Oa*-TAm) was combined in a cascade with an α-TAm to aminate α-keto acids for the production of unnatural amino acids. A number of bioactives, such as the ACE inhibitor perindopril and the antidiabetic saxagliptin, have been synthesized in this way, as well as intermediates for antibiotics and HIV drugs.

Chains consisting of several rounds of homologous sequence searches can be followed, such as that beginning with *Mz*-TAm, discovered following an activity-guided approach using enrichment media. Using *Mz*-TAm as a template, a β-TAm from *Polaromonas* sp. JS666 (*Po*-TAm) was discovered (Bea et al. 2011). In recent work by Kim and co-workers (Kim et al. 2019a, b), *Po*-TAm was used as a template, yielding eight novel TAms, among them an enzyme from *Ilumatobacter coccineus* (*Ic*-TAm). Within this same study, *Ic*-TAm was then used as a template in homology searches, yielding a further eight novel TAms for characterization (Table 1) (Kim et al. 2019a). This example highlights the powerful and iterative nature of mining sequence databases for putative sequences, followed by characterization and feeding this data back into the search engine, enhancing knowledge of sequence-function relationships and improving future searches.

**Fig. 2:**
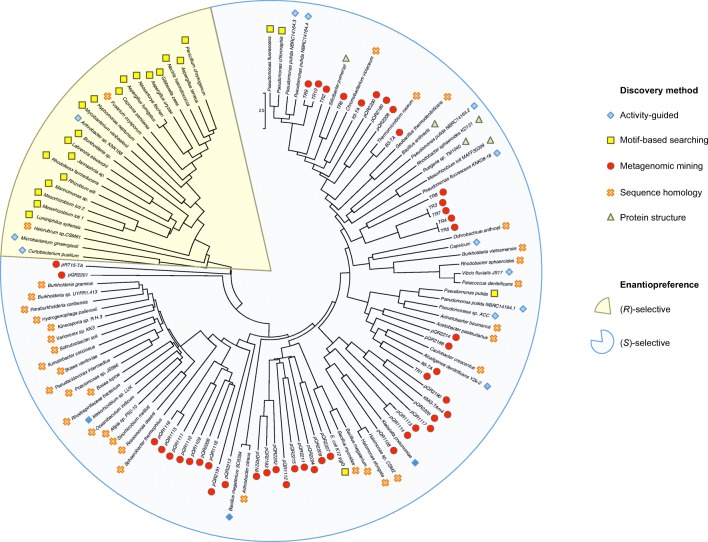
Phylogenetic tree highlighting evolutionary relationships between characterized TAms, categorized by method of discovery and enantiopreference. A neighbor-joining tree was produced using MEGA v. 7.0.26 with bootstrap values of 1000, following protein sequence alignment by ClustalW

While each iteration of homologous sequence searching uncovers novel sequences for putative enzymes, using sequence similarity as the sole basis for biocatalyst mining has some key drawbacks. As the search is based on overall sequence similarity, “similar” enzyme hits are indeed what tends to be found. This can be observed in a number of examples in the phylogenetic tree presented in Fig. 2, where newly discovered TAms are quite closely related to the query sequences from which they were found. This can be also seen in the example above, with *Po*-TAm and *Ic*-TAm used as search sequences. All of the novel TAms discovered during these searches exhibited relatively close phylogeny to their “parent” enzymes, and each other, all residing in the same clade within the tree. Similar examples can be seen with *Pd*-TAm (found based on homology to *Vf*-TAm) and a TAm from *Caulobacter crescentus* (Hwang et al. 2008) (found based on homology to *Ad*-TAm). This also means that very often the novel enzymes discovered are functionally quite similar to what already exists in the TAm toolbox, and more distantly related enzymes, which may have very useful or unexpected biocatalytic characteristics, are missed by the search. Furthermore, if we select enzymes whose similarity is too low, rather than being novel examples, may not be functional as TAms at all. This has, in part, led to alternative approaches in the search for novel, functional TAms using in silico search methods, or searching in previously untapped or extreme environments for new enzymes.

### Expanding the search to different environments

The majority of early TAms were discovered from mesophilic microorganisms, with many derived from soil bacteria. This is unsurprising given that these organisms are among the most well-characterized in microbiology as a whole. Furthermore, they are among the most widely sequenced; therefore, “hits” from these organisms were more likely following sequence searching. However, given the relatively benign environments from which these organisms are derived, many of their enzymes are unable to tolerate the harsh reaction conditions often demanded by industrial processes, such as high temperature and presence of organic solvents. In order to find wild-type enzymes which can tolerate these conditions, a number of research groups have recently begun mining the genomes of microorganisms isolated from extreme environments, or extremophiles.

Using *Vf*-TAm as query sequence, Chen et al. (2016) found a TAm in the genome of the thermophilic bacterium, *Geobacillus thermodenitrificans* (*Gb*-TAm). Despite using a sequence from a mesophilic bacterium as a template, by mining a microorganism from an extreme environment, the authors found an enzyme with optimum activity at 65 °C and pH 9, potentially useful properties for an industrial biocatalyst (Chen et al. 2016). Again using *Vf*-TAm as a template, Mathew and co-workers (Mathew et al. 2016a) discovered a novel (*S*)-TAm from the thermophilic bacterium *Thermomicrobium roseum*, which had optimum activity at 80 °C, as well as tolerance to a range of organic solvents (Mathew et al. 2016a). Work by the same group uncovered another thermophilic TAm from *Sphaerobacter thermophilus* (*St*-TAm), this time using *Po*-TAm as a query sequence, somewhat paradoxical given the low-temperature adaptations of *Polaromonas* sp. JS666, from which it is derived. *St*-TAm exhibited superior thermostability and activity at higher temperatures (60 °C) than the *Po*-TAm enzyme used to discover it, highlighting the benefits of interrogating the genomes of extremophiles in the search for robust enzymes (Mathew et al. 2016b).

The genomes of halophilic microorganisms have also been explored for TAms using similar methods. The Paradisi group characterized the first ω-TAm from a halophilic bacterium following a homologous sequence search with *Vf*-TAm. The resultant TAm from *Halomonas elongata* showed tolerance to organic solvents and optimum activity at pH 10 (Cerioli et al. 2015). A similar enzyme, Ad2-TAm, was characterized from the genome of *Halomonas* sp. CSM-2, a moderately halophilic bacterium isolated from a Triassic period salt mine. Ad2-TAm displayed no loss of function up to 1.5 M NaCl and organic solvent tolerance, as well as the ability to function in a seawater reaction medium (Kelly et al. 2017, 2019b).

**Fig. 3:**
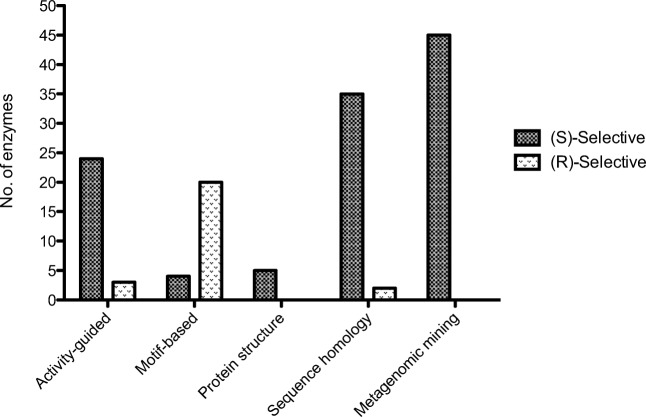
Comparison of the numbers of reported *R*- and *S*-selective TAms by discovery method from 1997 to 2019

Given the relative dearth of *R*-selective TAms uncovered using activity-guided approaches, it is not surprising that the number of characterized *R*-selective enzymes found using homology searches is much lower than their *S*-selective counterparts. Indeed, many *R*-selective TAms discovered using overall sequence homology appear to come from microorganisms other than bacteria. Gao et al. (2017) characterized an *R*-selective TAm from the fungus *Fusarium oxysporum* (Gao et al. 2017), while the first haloarchaeal TAm characterized for use in biocatalysis (BC61-TAm) was isolated from the genome of *Halorubrum* sp. CSM-61 (Kelly et al. 2019a). These examples reinforce the benefit of mining genomes from previously untapped or extreme environments, with both yielding relatively scarce and valuable *R*-selective TAms. The numbers of *S*- and *R*-selective enzymes discovered by different approaches are shown in Fig. 3.

### Discovery using key motifs and protein structure

As highlighted, one of the drawbacks of homologous sequence searching is the return of similar proteins. As more was understood about structure-function relationships, it became apparent that the search for novel TAms could center around finding key motifs, known to be important for function, rather than overall sequence similarity. By identifying key motifs and active site residues for function prediction in PLP fold type IV enzymes, Höhne and co-workers developed an in silico strategy for sequence-based prediction of substrate specificity and enantiopreference. Using this approach, 17 novel *R*-selective TAms were discovered from databases containing proteins of cultured organisms (Höhne et al. 2010). Interestingly, many of the *R*-selective TAms identified using this approach were derived from the genomes of fungi. The TAms from this study have already found applications in the asymmetric synthesis of pharmaceuticals, with one enzyme from *Aspergillus fumigatus* combined in a cascade with acetohydroxyacid synthase I in the synthesis of the decongestant norpseudoephedrine (Sehl et al. 2013). Similar studies have yielded further *R*-selective TAms from fungi, including *Cordyceps*, *Nectria*, *Capronia*, and *Trichoderma* spp. (Sayer et al. 2014; Jiang et al. 2015; Iglesias et al. 2017). Recent work saw yet another *R*-selective TAm isolated using the same approach, this time from *Actinobacter* sp., a bacterium which shares a number of characteristics with fungi (Tang et al. 2019).

As can be seen from these examples, and more broadly in Fig. 3, the majority of *R*-selective TAms to date have been discovered using key motif searches from cultured microorganisms, in particular from fungi. Profiling *R*-selective TAms in this way and highlighting discovery trends can help inform future mining strategies, which may be particularly useful given the relative scarcity of *R*-selective TAms in the literature at present.

As well as database searching using key motifs, searches using overall protein structure rather than sequence have been employed for TAm discovery. This approach has been driven largely by the Höhne group, resulting in a number of *S*-selective TAms (Steffen-Munsberg et al. 2013; Steffen-Munsberg et al. 2016).

## Metagenomic mining for enzyme discovery

### Sequence-driven metagenomics

Exploiting the riches of cultured microorganisms has been the mainstay of TAm discovery from the outset, firstly with activity-guided approaches, progressing to database mining through sequence homology, key motif, and protein structure searches. The drawback of using this approach, however, is that it can only utilize those microorganisms which can be cultured. As well as the time and effort required in isolation and DNA sequencing of individual genomes, it has been suggested that as few as 1% of microorganisms can be cultured in the laboratory (Rappé and Giovannoni 2003; Schloss and Handelsman 2004). This leaves the vast majority of microorganisms, and the enzymes encoded therein, inaccessible using culture-based techniques alone. Isolating DNA directly from the environment (eDNA) negates the need for culture and provides access to a far greater breadth of sequences within a given environment.

Metagenomic mining is still in its infancy, with relatively few studies compared to those from cultured organisms. This trend appears to be changing however, with the number of TAms discovered by metagenomic mining seeing a sharp rise in recent years (Fig. 1). TAms discovered using metagenomic mining are shown in Table 2.

**Table 2:** Summary of TAms discovered using metagenomic approaches

Enzyme	(*S*)/(*R*)	Comments	Reference
Sequence-driven approach			
pQR1108–pQR1118	(*S*)	Metagenome derived from oral cavities of humans	Baud et al. (2017)
Is3-TA	(*S*)	Metagenome derived from hot spring metagenomes in Italy and Iceland	Ferrandi et al. (2017)
It6-TA	(*S*)		
B3-TA	(*S*)		
pQR2188–pQR2191	(*S*)	Metagenome derived from DNA isolated from domestic drain	Leipold et al. (2019)
pQR2193	(*S*)		
pQR2200–pQR2202	(*S*)		
pQR2204–pQR2209	(*S*)		
pQR2211	(*S*)		
pQR2213–pQR2213	(*S*)		
KMG-TAm4	(*S*)	Metagenome derived from Triassic period salt mine	Kelly et al. (2018a, b)
Functional metagenomics			
pRT15-TA	(*S*)	Fosmid library generated for screening Sequence 15% shorter than *Vf*-TAm and *Cv*-TAm	Pawar et al. (2018)
TR_1_ to TR_10_	(*S*)^a^	Fosmid library	Coscolín et al. (2019)
		Metagenomic derived from 28 geographically distinct environments, including chronically polluted marine sediment samples, an acidic beach pool and the genome of *Pseudomonas oleovorans*	

^a^Enzymes TR_2_, TR_6_, TR_9_, and TR_10_ accepted both enantiomers of 2-aminononane

In one such study by the Hailes and Ward, 11 putative TAms were cloned and overexpressed from a metagenome obtained from the oral cavities of humans (Baud et al. 2017). An in silico sequence-driven approach was used to find TAm-encoding sequences, with the Pfam ID for Class III TAms used to extract open reading frames. The resultant sequences were cloned and overexpressed using standard molecular biology techniques and screened against a wide range of amino donors and carbonyl substrates. This represents a high throughput and targeted method for identifying putative TAm sequences from eDNA obtained via culture-independent means.

The same group used a similar sequence-driven approach to expand on this work, with a highly successful study focusing on the retrieval of TAms from a domestic drain metagenome (Leipold et al. 2019). Of the 36 full-length, non-redundant Class III TAm sequences found, 29 were successfully cloned and expressed in *Escherichia coli*. Percentage identities ranged from 16 to 36% compared to the well-characterized *Vf*-TAm and 17–54% with *Cv*-TAm, highlighting the novelty which can be achieved using this approach. The evolutionary diversity in the TAms from this study can be visualized in Fig. 2, with sequences well distributed right throughout those of known *S*-selective TAms (enzymes from this study were given the designation pQR2189 to pQR2216). In a substantial undertaking of characterization, these enzymes were screened for activity against a variety of amino donors and substrates, under a range of temperatures, pH values, and organic solvent concentrations. Perhaps the most promising enzyme, pQR2189, displayed the desirable ability to accept the amino donor isopropylamine (IPAm), as well as functioning impressively in up to 50% DMSO. What is arguably the most important conclusion from this study in terms of TAm discovery is the benefits to be garnered by combining a metagenomic mining approach with an unusual ecological niche, such as the largely uncharacterized domestic drain metagenome.

Extreme environments have also been mined for TAms using sequence-driven metagenomics, most notably from hot spring metagenomes from Iceland and Italy (Ferrandi et al. 2017). A number of *S*-selective TAms were discovered via alignment with query sequences. Despite these query sequences being of mesophilic origin, by virtue of searching in an extreme environment, a number of highly thermophilic TAms were found. One enzyme in particular, B3-TA, was found to be particularly thermostable, retaining 85% activity following incubation at 80 °C for 5 days, with optimum activity observed at 90 °C. Unsurprisingly, when the sequence for B3-TA is aligned against other characterized TAms from the literature, its closest relative is a TAm from the thermophile *T. roseum*, discussed previously.

Recent metagenomic mining from a hypersaline environment also yielded a functional TAm, KMG-TAm4 (Kelly et al. 2018b). In this case, however, the enzyme produced did not exhibit a halophilic or halotolerant activity profile, as had been observed in previous enzymes characterized from cultured microbes from the same source (Kelly et al. 2017, 2019a). This may be due to the adaptive mechanisms employed by some halophiles, where the organism as a whole is adapted to hypersaline environments by excluding salt from the cell, meaning intracellular enzymes do not themselves need to be salt-adapted. This also presents a cautionary tale, that even though a source environment may be extreme, not every enzyme represented in its metagenome will exhibit similarly extreme properties, given the diversity of mechanisms of extreme environment physiological adaptations exhibited by microorganism colonizing a given environmental niche.

It is worthy of note that all TAms found using sequence-derived metagenomics are *S*-selective. This is due largely to the search criteria used in enzyme discovery, with similarity to Class III TAms (which are *S*-selective) sought in studies by the Hailes and Ward. However, in the studies by Ferrandi et al. (2017) and Kelly et al. (2018b), PLP fold type IV enzymes were used as query sequences, but in either case, no functional *R*-selective TAms were uncovered. While this is a fairly small sample size and *S*-selective enzymes are more abundant naturally, it does perhaps highlight the need to use enhanced search techniques in the quest for novel *R*-selective enzymes from metagenomes, such as key motif-based searches which proved so successful with cultured microorganisms.

### Functional metagenomics

Due to the breadth of sequences which can be accessed through metagenomic mining of eDNA, many novel sequences which are distantly related to known enzymes, but still functional, can be uncovered. As with mining the genomes of cultured microbes, a sequence-driven approach still has limitations however, as searching using query sequences will still, in the main, provide us only with enzymes which are similar to those already characterized. As detailed earlier, activity-guided approaches, despite being constrained by culturability limitations, have the potential for discovery of enzymes which are very different to those already described, as they are not discovered based on sequence similarity to current enzymes, but on substrate turnover. To pursue activity-guided approaches from cultured microorganisms is time consuming and tedious work however, compounded by the inability to cultivate the vast majority of microorganisms in a lab setting. This can also result in over-mining an already restricted and limited resource, namely the 1% culturable portion of a given microbiome. Functional metagenomics could have a huge part to play in circumventing these shortfalls. This involves isolating eDNA, cutting it into pieces of a desired size range and cloning these pieces into expression vectors directly. Bacteria, usually *E. coli*, are transformed with the recombinant vectors and screened for activity. Alternatively, vectors containing a gene of interest, such as a TAm-encoding gene, are selected using enrichment media, similar to early activity-guided screens discussed above.

To date, functional metagenomic studies for TAms are scarce but have involved the generation of fosmid libraries for novel enzyme discovery. In one such approach, metagenomic fosmid libraries from 28 geographically distinct environments were produced and screened using well-established agar-based colorimetric assays (Green et al. 2014; Baud et al. 2015) to show clones harboring relevant TAm-encoding sequences (Coscolín et al. 2019). Analysis of candidates at protein sequence level revealed large divergence among the Class III TAms identified, with no particular type of protein dominant.

Of the 10 enzymes characterized, all were *S*-selective. Interestingly, however, 4 of these enzymes (TR_2_, TR_6_, TR_9_, and TR_10_) were able to accept both the *S*- and *R*-enantiomers of 2-aminononane. Unlike the other Class III TAms uncovered by this screen, these enzymes all had a large pocket volume, a hairpin region close to the conserved arginine residue at position 414 and an outward orientation of the Arg414 residue itself, although the authors postulate that other factors may be at play in determining the ability of these enzymes to accept both *S*- and *R*-substrates (Coscolín et al. 2019).

Using a similar approach of fosmid library generation and colorimetric screening using *o*-xylylenediamine as amino donor, Pawar and co-workers (Pawar et al. 2018) discovered a (*S*)-selective TAm with a high degree of novelty. pRT15-TA was < 30% similar to *Vf*-TAm and *Cv*-TAm, as well as being 15% shorter. The novelty of this enzyme can be seen in Fig. 2, with pRT15-TA occupying a position on its own branch, with considerable evolutionary distance from all other characterized TAms. Despite these differences in sequence, pRT15-TA was stably expressed in *E. coli* and capable of accepting a number of ketone and amine substrates, as well as being the first TAm to be discovered using a functional metagenomics approach (Pawar et al. 2018).

As with other discovery methodologies discussed, functional metagenomics is not without limitations. While colorimetric screens such as those employed by Coscolín et al. (2019) and Pawar et al. (2018) increase screening efficiency considerably, expression and screening of clones is not trivial and can be time consuming and resource heavy. Furthermore, not all enzymes can be easily expressed in established *E. coli*-based systems, such as those from extremophiles, whose characteristics can be very desirable. To date, expression systems for functional metagenomics of extremozymes are limited, with no examples of TAms as yet. Despite these drawbacks, a functional metagenomics approach provides access to a vast range of genes which can be screened for functional activity, including from microbes which cannot be cultured in the lab. As such, it is a powerful tool which looks set to feature more prominently in future enzyme discovery attempts.

## Protein engineering

While this mini-review focuses largely on the discovery of novel wild-type enzymes, it would be remiss not to include some important examples of engineered TAms, given the increasingly influential role rational design plays in TAm biocatalysis. As alluded to previously, protein engineering has facilitated some of the greatest success stories of TAm application in the pharmaceutical industry, none more so than the engineered variant of *Arth*-TAm, used in the synthesis of sitagliptin.

The number of studies involving protein engineering of TAms has continued to increase in recent years. Using an in silico design approach, seven mutations to the well-characterized *Vf*-TAm resulted in a 1716-fold increase in reaction rate towards the bulky ketone substrate, 2-acetylbiphenyl (Dourado et al. 2016). This focused mainly on enlarging the large binding pocket and increasing hydrophobicity at key residues in order to improve specificity and binding. An *S*-selective TAm from *Ruegeria* sp. TM1040 was also rationally designed for the synthesis of bulky chiral amines. The sequence of the evolved enzyme was used in the discovery of six new TAms with activity towards bulky substrates (Pavlidis et al. 2016). This demonstrates the cyclical and iterative nature of biocatalyst discovery and the value of combining different discovery approaches. Advances made with protein engineering can be fed back into the discovery pipeline in the quest for novel wild-type TAms, as well as new enzymes informing which mutations may be beneficial in future protein engineering. Similar studies have improved the activities of other enzymes through protein engineering such as *Oa*-TAm (Kim et al. 2019b) and *Cv*-TAm (Almahboub et al. 2018; Voss et al. 2018), as well as engineering a strain of yeast to contain *Cv*-TAm for application in whole cell biocatalysis (Braun-Galleani et al. 2019).

Other characteristics have been improved through protein engineering, such as enhanced thermostability of an *R*-selective TAm from *Aspergillus terreus* through the introduction of disulphide bonds (Xie et al. 2018). Indeed, rational design has even proved capable of changing the enantiopreference of a number of TAms. In one such example, the enantiopreference of *Cv*-TAm was changed from *S*- to *R*-selective by swapping phenylalanine and alanine residues at positions 88 and 231 (Humble et al. 2012). Furthermore, work by the Dalby group has shown that ω-TAm activity can be switched towards α-TAm activity via protein engineering (Deszcz et al. 2015).

As well as finding the enzymes of the future, rational design of TAms has been used to uncover ancestral proteins in a process described as “reverse engineering” (Wilding et al. 2017). Ancestral biocatalysts were found to be more promiscuous than their modern counterparts, with superior activity compared to their descendants. These results may also help to guide future wild-type enzyme discovery, suggesting ancient and unspoiled environments may harbor wild-type ancestral biocatalysts with naturally promiscuous substrate scopes and high activities.

The recently launched ω-Transaminase Engineering Database (oTAED) brings together protein structure and sequence for ω-TAms, classifying enzymes based on fold type and sequence similarity and applying a standard numbering scheme for equivalent residues in homologous proteins (Buß et al. 2018). Functional information is also attributed to residues where known, making this publicly accessible database a useful tool in future TAm engineering and discovery studies. The importance of crystal structures, and indeed good homology models, should not be underestimated in its ability to enhance databases such as oTAED, and therefore TAm protein engineering overall.

Protein engineering has a vital role in the development of new TAms with enhanced characteristics, not only in improving the individual enzyme in each case but also in advancing knowledge for future engineering and novel TAm discovery. Lessons learned from protein engineering studies inform future searches for wild-type TAms, just as novel TAms with desirable characteristics can provide a scaffold for protein engineering. As such, protein engineering continues to play a vital role in the field, enjoying a complementary relationship with wild-type TAm discovery.

## Concluding remarks and outlook

TAm discovery has changed and evolved since the inception of the field several decades ago, benefitting from improvements in sequencing technologies and burgeoning protein databases along the way. Early work focused heavily on culture-dependent, activity-guided approaches, often through the use of enrichment media to find microbes displaying a desired phenotype. As sequencing became more cost-effective and accessible, increasing numbers of TAm sequences became available. This enabled the mining of vast repositories of whole genome data for homologous sequences, giving rise to many of the best characterized TAms in use today.

As more enzymes were discovered and characterized using both activity-guided and sequence homology approaches, knowledge of sequence- and structure-function relationships was improved, allowing key motifs for activity to be identified. This enabled more targeted database mining of novel TAms, facilitating the discovery of many new *R*-selective TAms in particular.

The advent of metagenomic mining looks set to exert a significant effect on the field, providing access to vast amounts of previously inaccessible sequence data. The effects of this change, while recent, are already being seen, with large numbers of enzymes discovered and characterized already from a relatively small number of studies.

Much remains unknown about TAms, with the function of many conserved residues still to be deciphered. While in silico enzyme analysis becomes increasingly sophisticated, wet lab experiments are still required for definitive characterization of novel enzymes and engineered mutants. In future, the field is likely to benefit from advances in machine learning, with iterative cycles of mining, engineering, testing, and feedback improving knowledge of structure-function relationships and enhancing algorithms for further discovery. Continued discovery and characterization of new wild-type enzymes with desirable properties could also feed into these pipelines, helping to provide a scaffold for biocatalyst design.

TAm biocatalysis has benefitted hugely from all approaches employed in enzyme discovery, with each approach improving the knowledge base of the field and informing future discovery. Activity-guided approaches, in particular through functional metagenomic screens of previously untapped ecological niches, are likely to increase in the coming years. This, combined with protein engineering and searches based on sequence and structure, looks likely to continue to shape the near future of novel TAm discovery for industrial biocatalysis.
